# Threshold-based evolutionary magnitude estimation for an earthquake early warning system in the Sichuan–Yunnan region, China

**DOI:** 10.1038/s41598-020-78046-2

**Published:** 2020-12-03

**Authors:** Yuan Wang, Shanyou Li, Jindong Song

**Affiliations:** Institute of Engineering Mechanics, China Earthquake Administration, Key Laboratory of Earthquake Engineering and Engineering Vibration of China Earthquake Administration, Harbin, 150080 China

**Keywords:** Solid Earth sciences, Seismology

## Abstract

The Sichuan–Yunnan region is one of the most seismically vulnerable areas in China. Accordingly, an earthquake early warning (EEW) system for the region is essential to reduce future earthquake hazards. This research analyses the utility of two early warning parameters (τ_c_ and P_d_) for magnitude estimation using 273 events that occurred in the Sichuan–Yunnan region during 2007–2015. We find that τ_c_ can more reliably predict high-magnitude events during a short P-wave time window (PTW) but produces greater uncertainty in the low-magnitude range, whereas P_d_ is highly correlated with the event magnitude depending on the selection of an appropriate PTW. Here, we propose a threshold-based evolutionary magnitude estimation method based on a specific combination of τ_c_ and P_d_ that both offers more robust advance magnitude estimates for large earthquakes and ensures the estimation accuracy for low-magnitude events. The advantages of the proposed approach are validated using data from 2016–2017 and the Ms 8.0 Wenchuan earthquake in an offline simulation. The proposed concept provides a useful basis for the future implementation of an EEW system in the Sichuan–Yunnan region.

## Introduction

Earthquake early warning (EEW) systems offer promise as a practical tool in seismic hazard mitigation that could help reduce the losses caused by destructive earthquakes. Using data from a dense instrument array around the source region or near the target of concern, EEW systems aim to broadcast advance warning of potential damage from an ongoing earthquake to target areas before the arrival of destructive seismic waves^1^. Following several decades of development, EEW systems are currently operational and provide alerts to the public in several countries or regions worldwide^2^, including Japan^3^, Taiwan^4^, Mexico^5^ and South Korea^6^; in contrast, some EEW systems operating in India^7^, Romania^8^, Turkey^8^ and the West Coast of the United States^9^ issue alerts only to selected users. In addition, other EEW systems are currently under real-time testing or development in locations including Italy^10^, Chile^11^, Coast Rica^2^, EI Salvador^2^, Nicaragua^2^, Switzerland^2^, Israel^12^, and the Beijing capital region^13^ and Fujian region^14^ of China.

Two main approaches are adopted for EEW systems: regional warning (network-based) and onsite warning (stand-alone). In the onsite warning approach, the initial P-wave motion of a single station or local array is used to predict the subsequent peak ground shaking at the same site. In contrast, to issue a regional warning, data from seismic networks deployed in the potential source area are used to estimate the source parameters (location and magnitude) and predict the ground motion for the concerned region. The advantage of onsite warning is that it provides faster warnings of future ground motion to sites at short distances from the epicenter, commonly through setting a P-wave amplitude threshold without necessarily determining the location and magnitude of the event, whereas the regional warning approach provides a more robust ground motion prediction for a wider region with continuously available network data and source parameters updated in real time. The earliest EEW systems generally adopted the onsite warning method due to the limited network density. At present, following increases in network density, the onsite algorithm is employed in most network-based warning systems to integrate the advantages of the regional and onsite warning approaches, thereby providing more information and the quickest warnings.

The Sichuan–Yunnan region, located at the eastern edge of the Qinghai–Tibet Plateau, coincides with the middle to southern section of the north–south seismic zone that extends across the central part of the Chinese mainland. The high level of seismicity in this region is associated with its complex tectonic setting. The Sichuan–Yunnan region has experienced numerous destructive earthquakes that have caused extensive casualties and property losses; remarkable examples include the Ms 6.4 Ning’er earthquake^15^ on 3 June 2007, the Ms 7.0 Lushan earthquake^16,17^ on 20 April 2013, and the Ms 6.5 Ludian earthquake^18^ on 3 August 2014. Of particular note is the Ms 8.0 Wenchuan earthquake, which occurred on 12 May 2008 and resulted in more than 69,000 deaths and financial losses reaching hundreds of billions of RMB^19^. As the effects of potential earthquake hazards will continue to increase with the rising population and economy in this area, scientific research on diminishing future earthquake hazards is essential for the Sichuan–Yunnan region. In this context, to strengthen seismic prevention and disaster mitigation measures therein, the Sichuan–Yunnan region has been designated the key monitoring area of the Seismic Intensity Rapid Reporting and Earthquake Early Warning (SIRR & EEW) project, and developing an EEW system for this region is of great concern.

Rapidly estimating the magnitude of an earthquake event is the most critical obstacle to the widespread development of EEW systems and is typically achieved based on empirical scaling relationships between the magnitude and early warning parameters from initial P-waves. Research has confirmed that the average period (τ_c_), the most commonly used parameter for estimating the magnitude with regard to the P-wave frequency, is correlated with the earthquake magnitude^1,20^. In addition, the peak amplitude of the vertical displacement (P_d_) in the first few seconds of the P-wave has been shown to be related to the event magnitude and is used mainly as information about the amplitude content of early P-waves to predict the earthquake magnitude^21,22^. Models based on τ_c_ and P_d_ have been proposed for the EEW systems in China to estimate the earthquake magnitude using only sequence data for large events that occurred in the Sichuan–Yunnan region^23,24^. Fortunately, with the operation of the China Strong Motion Network Center (CSMNC), large quantities of high-quality digital strong motion data have been recorded, constituting a reliable database from which the relationships between the event magnitude and the EEW parameters τ_c_ and P_d_ can be established for the Sichuan–Yunnan region.

Previous studies have shown that τ_c_ typically exhibits large scatter in the low-magnitude range^25,26^, and the application of P_d_ to predicting the magnitude of large events is limited by the length of the P-wave time window (PTW)^27^. For the reliable and early assessment of destructive earthquakes, Wu and Kanamori^20^ originally proposed the integrated use of τ_c_ and P_d_ to establish a threshold-based EEW system, which was subsequently applied in various areas^26,28^. However, low signal-to-noise ratio (SNR) records from low-magnitude events may produce an unexpectedly large τ_c_ value^29^, in which case the direct combination of these two parameters with such an unexpected error may overestimate a small event, resulting in a false alarm.

**Figure 1:**
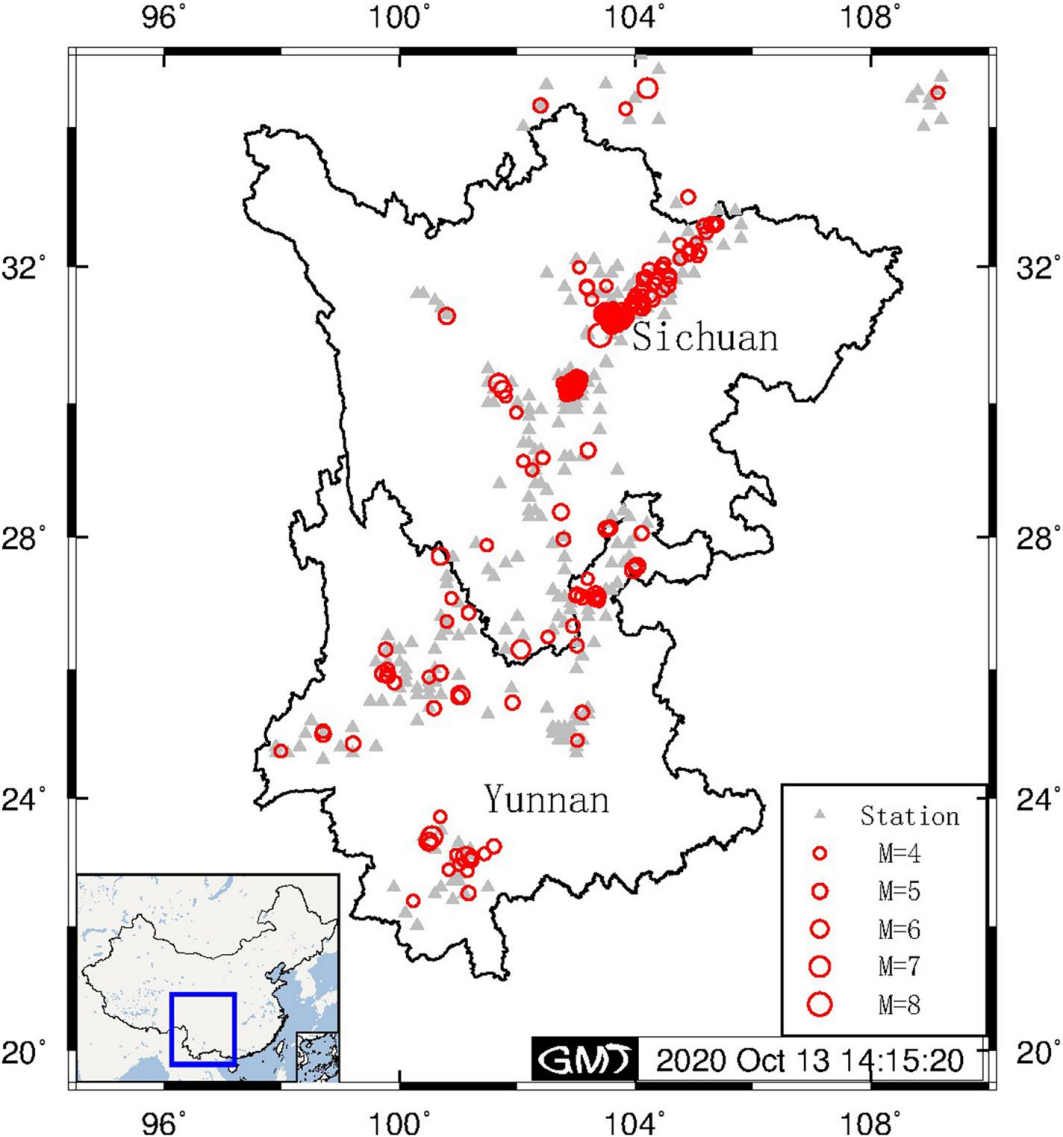
Event map. Epicenter locations of the 273 selected events (red open circles; the size is proportional to the magnitude) and the distribution of CSMNC stations (gray solid triangles) used in this study. The figure was made by using the Generic Mapping Tool (GMT) v.5.2.1 (www.soest.hawaii.edu/gmt).

**Figure 2:**
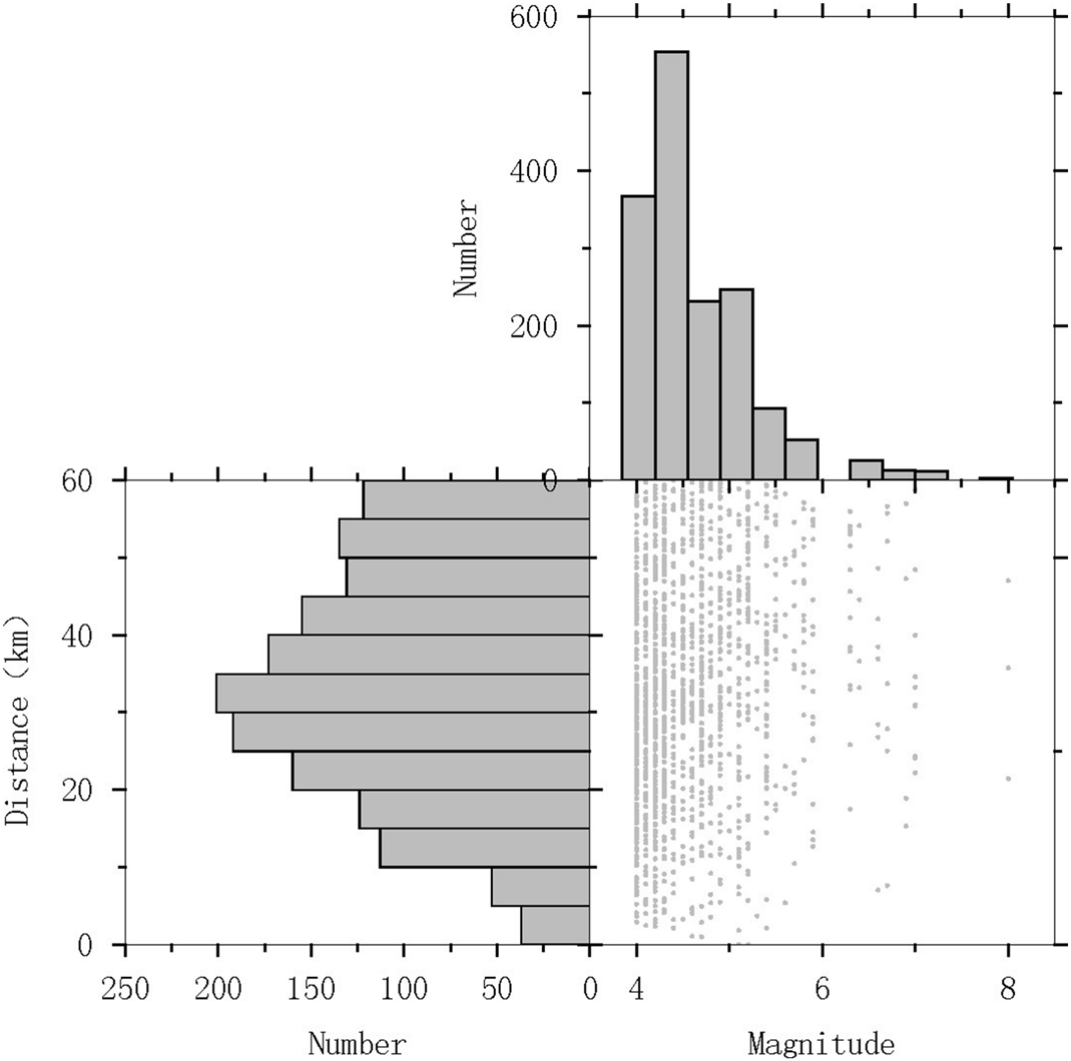
Data histogram. Distributions of the number of strong ground motion records in the fitting dataset as a function of magnitude and epicentral distance.

In this paper, we explored a similar magnitude estimation approach with a special combination of τ_c_ and P_d_. We proposed first judging whether τ_c_ should be combined with P_d_ to improve the magnitude estimation stability, and then we further explored the evolutionary magnitude estimation of the proposed approach. Here, we selected 1596 vertical components from 273 events that occurred within the Sichuan–Yunnan region during 2007–2015 as the fitting dataset for linear regression; furthermore, data from 13 events that occurred in the same region during 2016–2017 are employed as the validation dataset to evaluate the reliability of the proposed approach (Supplementary Table S1). The event magnitudes are in the range of 4.0–8.0, and all magnitudes (typically surface wave magnitudes) are denoted M. Since the high-frequency direct body waves radiating from a crustal earthquake rupture dominate in amplitude within the near-source range^21^ and the records of near-source stations represent the earliest information for rapid assessment and EEW, only records from stations with an epicentral distance of less than 60 km are considered. To ensure good station coverage for each event, at least three strong motion records for each earthquake must be available. The locations of the epicenters and CSMNC strong motion stations of the fitting dataset are shown in Figs. 1, and 2 illustrates the distribution of the strong motion records within the fitting dataset as a function of both their epicentral distance and their magnitude.

## Results

### Magnitude relationships with τ_c_ and P_d_

**Figure 3:**
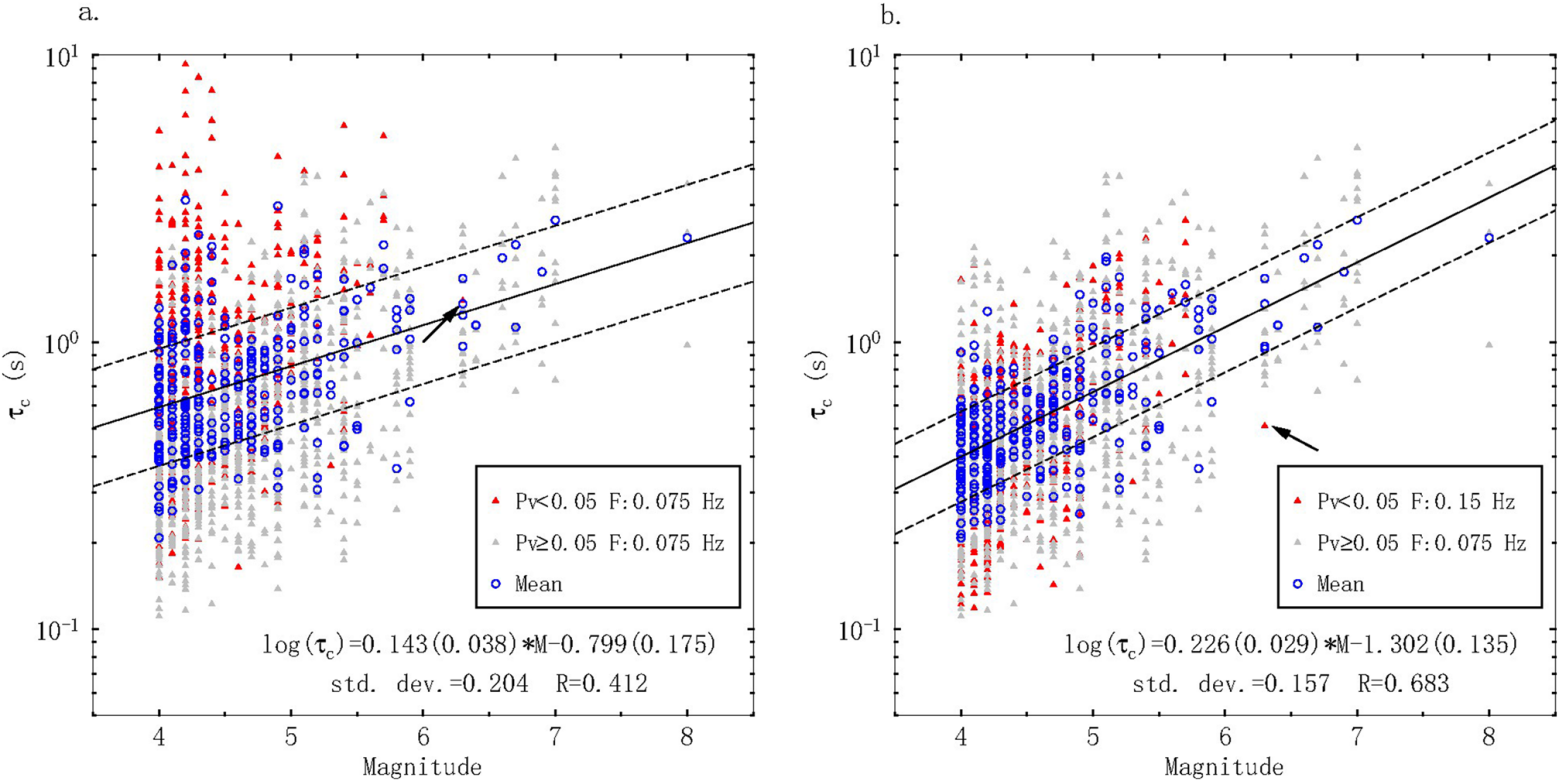
Comparison of the τ_c_-magnitude scaling relationships from the first 3 s of the PTW between the a) unapplied and b) applied criterion for restricting low-SNR data. τ_c_ values of individual records with P_v_ < 0.05 cm/s and P_v_ ≥ 0.05 cm/s are shown by red and gray triangles, respectively. Blue circles represent the mean τ_c_ value of each event. The solid line denotes the fitting relationship, and the dashed lines refer to ± 1 standard deviation. The record of the single M > 6 earthquake with Pv < 0.05 cm/s is marked with a black arrow.

We calculated τ_c_ from the vertical components of the strong motion records in the fitting dataset to investigate the relationship between τ_c_ and M. As illustrated in Fig. 3a, τ_c_ exhibits a linear correlation with the event magnitude and can effectively represent the scale of earthquakes. However, we found high τ_c_ values in the low-magnitude range for some records, resulting in large dispersion for events with M < 5.5. These abnormal τ_c_ values are usually affected by low-frequency drift of the integrated displacement with low-SNR or low-amplitude data, which may introduce large errors in τ_c_ and lead to false alarms. Following Zollo et al.^16^, we found that P_v_ (the peak amplitude of the velocity for a PTW of 3 s) reliably represents low-SNR data and is closely related to the displacement drift during the computation of τ_c_. Accordingly, we set a threshold of P_v_ = 0.05 cm/s to identify low-SNR waveforms and found that P_v_ < 0.05 cm/s typically corresponds to low-magnitude events with abnormally high τ_c_ values (Fig. 3a). Hence, to improve the stability of τ_c_, when P_v_ < 0.05 cm/s, we applied a stronger high-pass filter with a cutoff frequency of 0.15 Hz (increased from 0.075 Hz) to the integrated displacement. Only one earthquake record with a magnitude larger than 6 in our dataset features P_v_ < 0.05 cm/s, as highlighted by the arrow in Fig. 3a, indicating that our threshold (P_v_ = 0.05 cm/s) is small enough that the stronger filter (0.15 Hz high-pass filter) will rarely be applied to large events, thereby avoiding the loss of considerable low-frequency components.

After applying this criterion to restrict low-SNR data, the best-fit relationship between the magnitude and mean τ_c_ value of each event for PTW = 3 s was established, and the result is illustrated in Fig. 3b.

1 $$log_{10} \left( {\tau_{c} } \right) \, = \, 0.226(0.029)M - 1.302\left( {0.135} \right) \pm 0.157$$

The magnitude is estimated as follows.

2 $$M = \, 4.425log_{10} \left( {\tau_{c} } \right) \, + 5.761 \pm 0.694$$

The improved results are depicted in Fig. 3. The application of this criterion constrained all abnormally high τ_c_ values and resulted in scaling τ_c_ well with the magnitude up to M = 8. In addition, the correlation coefficient of this relationship increased from 0.412 to 0.683. According to our magnitude estimation equation [Eq. (2)], although the low slope (0.226) of the regression line still results in a large deviation of 0.694 magnitude units across the entire magnitude range, the τ_c_ values exhibit good scaling with large event magnitudes (M > 6.5) within acceptable error.

Next, we calibrated the P_d_ attenuation relationship and established a correlation between the distance-corrected P_d_^10km^ and magnitude for every PTW within 2–10 s (see Methods section). The calibrated coefficients are summarized in Supplementary Table S2. As an example, for PTW = 3 s, the following best-fit regression and magnitude estimation equations were obtained.

3 $$log_{10} (P_{d}^{10km} ) \, = \, 0.568M - 3.842 \pm 0.263$$

4 $$M = \, 1.761log_{10} (P_{d}^{10km} ) \, + 6.764 \pm 0.463$$

**Figure 4:**
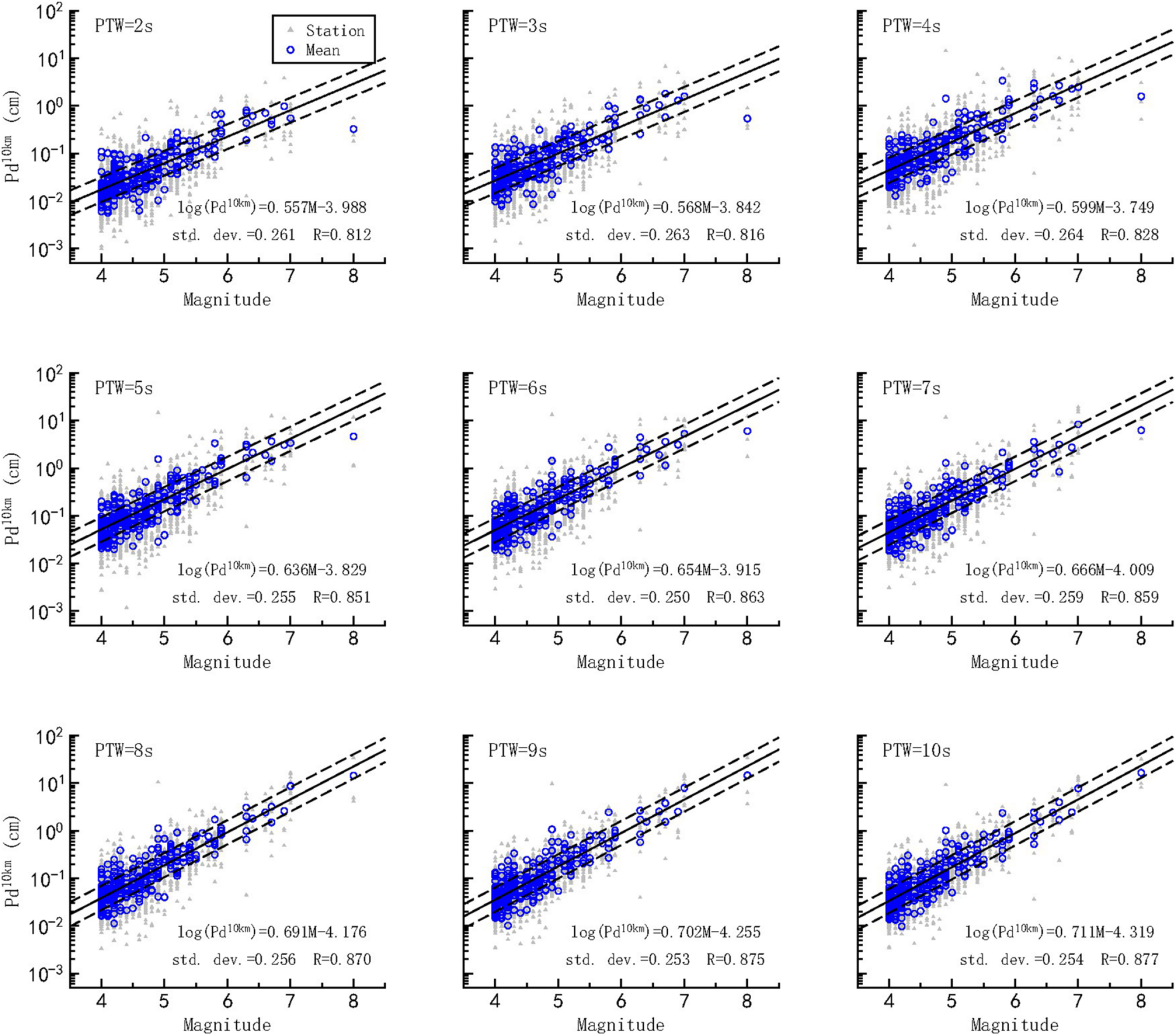
P_d_^10km^-magnitude scaling relationships for PTW = 2–10 s. The P_d_^10km^ values of individual records are denoted by gray triangles, and the averaged P_d_^10km^ values are denoted by blue circles. The solid line indicates the fitting relationship, and the dashed lines indicate ± 1 standard deviation.

Our results show that P_d_^10km^ achieved a deviation of 0.463 magnitude units, which is smaller than that of 0.694 magnitude units achieved by τ_c_ for PTW = 3 s. However, the low P_d_^10km^ values obtained for the M = 8 event deviate considerably from the expected relationship in the first 2–3 s (Fig. 4); this is consistent with previous studies, which have shown that P_d_ underestimates the magnitudes of large events for short PTWs^27,30^. In our study, P_d_^10km^ for the M = 8 event increased with increasing PTW, and the magnitude saturation phenomenon disappeared gradually when the PTW reached 8 s. Over the PTW range considered herein, the magnitude deviations of all the events changed from 0.469 magnitude units (PTW = 2 s) to 0.357 magnitude units (PTW = 10 s) (Supplementary Table S2).

### Characteristics of τ_c_ and P_d_^10km^ over different magnitude and PTW ranges

Based on the established magnitude scaling relationships for τ_c_ and P_d_^10km^, we computed the estimated magnitude of each event by using the mean value of both parameters. To investigate the general performance of each parameter in different magnitude ranges, we averaged the magnitude estimates of the binned events and compared them with the catalog magnitude. The interval of the bin was set to 0.1 magnitude units (ΔM = 0.1) to avoid obscuring the difference within each bin. Moreover, the standard deviation of the magnitude estimates was also given to show the discreteness of the magnitude estimates.

**Figure 5:**
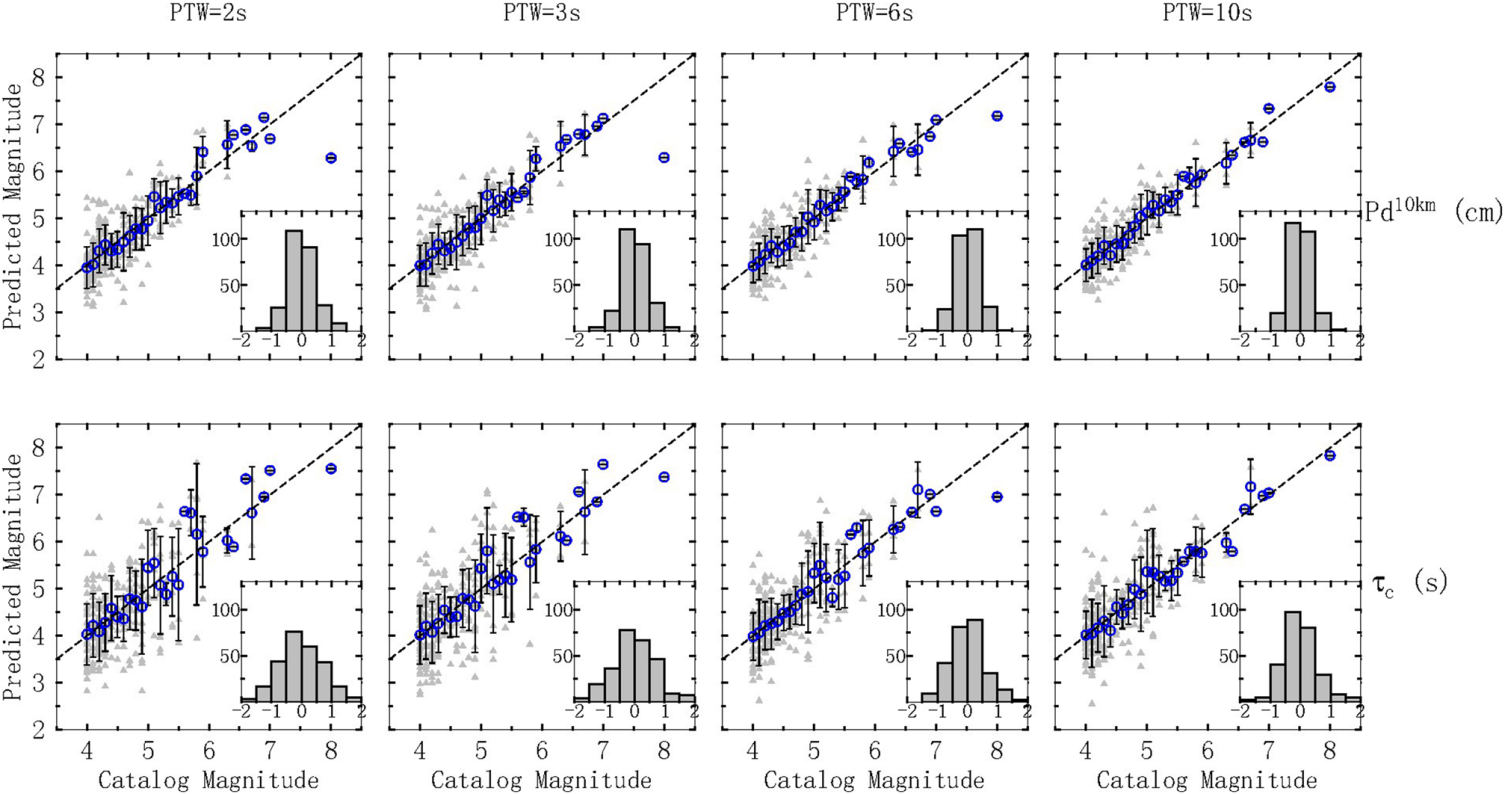
Catalog magnitude and magnitudes estimated by τ_c_ and Pd^10km^ at different PTWs. The dashed line represents 1:1 relationship. Grey triangles represent the predicted magnitude of each event. The blue squares represent the mean values of the estimates computed using average binned data (ΔM = 0.1). The standard deviations of the predicted magnitude using average binned data (ΔM = 0.1) are shown by black lines. Each subplot shows the estimated error distribution for small-moderate events (M < 6.5).

For a short PTW of 3 s, the P_d_^10km^-magnitude relationship established above achieve a correlation coefficient of 0.815, which is larger than the coefficient of 0.683 from the τc-magnitude relationship, indicating that P_d_^10km^ shows a higher magnitude correlation for PTW = 3 s compared to τ_c_ over the magnitude range from 4 to 8. As Fig. 5 indicates, for small-moderate events (M < 6.5), P_d_^10km^ predicts the magnitude with a standard deviation of approximately 0.40 magnitude units for each bin, whereas most standard deviations of τ_c_ are larger than 0.6 magnitude units. To clearly depict the performance of each parameter, we also provide subplots of the estimated error distributions for small-moderate events (M < 6.5) in Fig. 5, showing that 76.4% of all events have an estimated error of less than 0.5 magnitude units using P_d_^10km^ compared with 54.3% using τ_c_. Furthermore, the estimated errors of P_d_^10km^ for these events are all within 1.5 magnitude units, whereas 12 events exceed this value for τ_c_. In contrast, for the large-magnitude range, τ_c_ shows a smaller underestimation error (0.6 magnitude units) when estimating the largest event (the Ms 8.0 Wenchuan earthquake) compared to P_d_^10km^ (1.7 magnitude units).

We further studied the changes in the τ_c_ and P_d_^10km^ magnitude estimates with an extended PTW. For P_d_^10km^, with PTW increasing from 2 to 10 s, the percentage of events with an estimated error within 0.5 magnitude units gradually increases from 74.9 to 84.2%. Although the correlation coefficient increases only from 0.812 to 0.877, the underestimation error for the largest events at PTW = 2 s (1.7 magnitude units) shows obvious improvement and eventually reaches an underestimation error of 0.2 magnitude units once the PTW reaches 10 s. We also explored the effect of increasing the PTW on τ_c_. As shown in the bottom row of Fig. 5, the τ_c_-estimated magnitude error in the small-moderate magnitude range gradually decreases as the PTW is extended, but the discreteness of τ_c_ is still greater than that of P_d_^10km^ under the same PTW. Moreover, we found that τ_c_ exhibits excellent performance in predicting the largest M = 8 events with an underestimation error of 0.2 magnitude units when PTW = 10 s, but unlike the consistent, stable improvement of P_d_^10km^ in predicting large magnitudes with increasing PTW, the underestimation error by using τ_c_ increases from 0.6 to 1.0 magnitude units when PTW extends from 3 to 6 s.

Thus, the magnitudes of large events can be predicted by τ_c_ with an acceptable error using only 2 s of data after the P-wave arrival, and P_d_^10km^ is robust in predicting small-moderate event magnitudes. In addition, τ_c_ results in larger dispersion than P_d_^10km^ in predicting small-moderate earthquake magnitudes. This is the result after applying the criterion restricting low-SNR data; otherwise, the dispersion would be much larger. Ultimately, with increasing PTW, the saturation of P_d_^10km^ in predicting large event magnitudes can be obviously improved, while the improvement of τ_c_ is not consistent, and more data are needed for validation.

### Threshold settings for τ_c_ and P_d_^10km^

Based on the above characteristics of τ_c_ and P_d_^10km^ over different magnitude and PTW ranges, we proposed a threshold-based evolutionary magnitude estimation method that suggests the use of P_d_^10km^ to predict events within a wide magnitude range and jointly using τ_c_ to reduce the underestimation error only for large events.

The thresholds of τ_c_ and P_d_^10km^ were determined to discriminate large events (M > 6.5). To avoid excluding large earthquakes, we selected − 1σ standard deviation, and we set the τ_c_ and P_d_^10km^ thresholds corresponding to M = 6.5 to be 1.018 s and 0.387 cm, respectively, for PTW = 3 s (Supplementary Fig. S1). The threshold corresponding to M = 6.5 for every PTW considered for P_d_^10km^ is presented in Supplementary Table S3. According to a comparison between the real-time calculated parameters and the established thresholds, four potential situations are encountered: (1) τ_c_ > threshold and P_d_^10km^ > threshold; (2) τ_c_ > threshold and P_d_^10km^ < threshold; (3) τ_c_ < threshold and P_d_^10km^ > threshold; and (4) τ_c_ < threshold and P_d_^10km^ < threshold. We defined a four-entry decision table corresponding to the above situations: (1) the event is most likely to be a large earthquake; (2) the event may be a large earthquake with a small P_d_^10km^ caused by a short PTW or a small-moderate earthquake with an unexpectedly large τ_c_; (3) the event may be a large earthquake with an unexpectedly small τ_c_ or a small-moderate earthquake with a large P_d_^10km^; and (4) the event is most likely to be a small-moderate earthquake.

### Evolutionary magnitude estimation

As more information is needed for situations (2) and (3) to discriminate large events and the PTW needs to be extended to estimate larger-magnitude events by P_d_^10km^, the conditions under which to extend the PTW must be determined. To maximize the timeliness of our method and reduce the risk of missing large events, our method stops extending the PTW at 3 s when both P_d_^10km^ and τ_c_ are below their respective thresholds [situation (4)]; otherwise, our method extends the PTW [situations (1)-(3)].

With a certain PTW, our approach implements different strategies to estimate the event magnitude from the observation of a single station by analyzing the four potential situations stated above. We jointly use τ_c_ to calculate the average magnitude only when both P_d_^10km^ and τ_c_ exceed their respective thresholds [situation (1)] and otherwise take the magnitude estimation of P_d_^10km^ as the magnitude estimate under any other set of conditions [situations (2)–(4)]. For situation (1), we introduced weights to compute the final magnitude estimate by combining the magnitude estimates from P_d_^10km^ and τ_c_. Unlike the typical weights established based on the standard error of magnitude relationships under the overall magnitude range, here, considering that the main purpose of situation (1) is to reduce the magnitude saturation of large events, we decided to compute the weights based on the underestimation error for M = 8 events according to the relationship established above under each fixed PTW, as presented in Supplementary Table S4. The weights of P_d_^10km^ and τ_c_ for PTW = *i* s and the magnitude estimate of a single station based on these weights was obtained as follows.

5 $$W_{{i\tau_{c} \left( {iPd^{{10\,{\text{km}}}} } \right)}} = \frac{{1/\sigma_{{i\tau_{c} \left( {iPd^{{10\,{\text{km}}}} } \right)}} }}{{1/\sigma_{{i\tau_{c} }} + 1/\sigma_{{iPd^{{10\,{\text{km}}}} }} }};\;M = W_{{i\tau_{c} }} *M_{{i\tau_{c} }} + W_{{iPd^{{10\,{\text{km}}}} }} *M_{{iPd^{{10\,{\text{km}}}} }}$$

where ${\sigma }_{\mathrm{i\tau c}(\mathrm{iPd}^{10\mathrm{km}})}$, $\text{W}_{\mathrm{i\uptau c}(\mathrm{iPd}^{10\mathrm{km}})}$ and $\text{M}_{\mathrm{i\uptau c}(\mathrm{Pd}^{10\mathrm{km}})}$ are the underestimation error of M = 8 events, the weights and the magnitude estimates of τ_c_(P_d_^10km^) for PTW = *i* s, respectively.

The final magnitude was computed based on the results of multiple triggered stations in a seismic network. Since numerous distant stations with short PTWs will tend to lower the estimates of the average magnitude, we obtained average values based on the length of the PTW for each station as follows.

6 $$M = \frac{{\sum\nolimits_{i = 1}^{n} {M_{i} \times PTW_{i} } }}{{\sum\nolimits_{i = 1}^{n} {PTW_{i} } }}$$

where M_i_ and PTW_i_ are the predicted magnitude and PTW, respectively, for the *i*-th triggered station.

**Figure 6:**
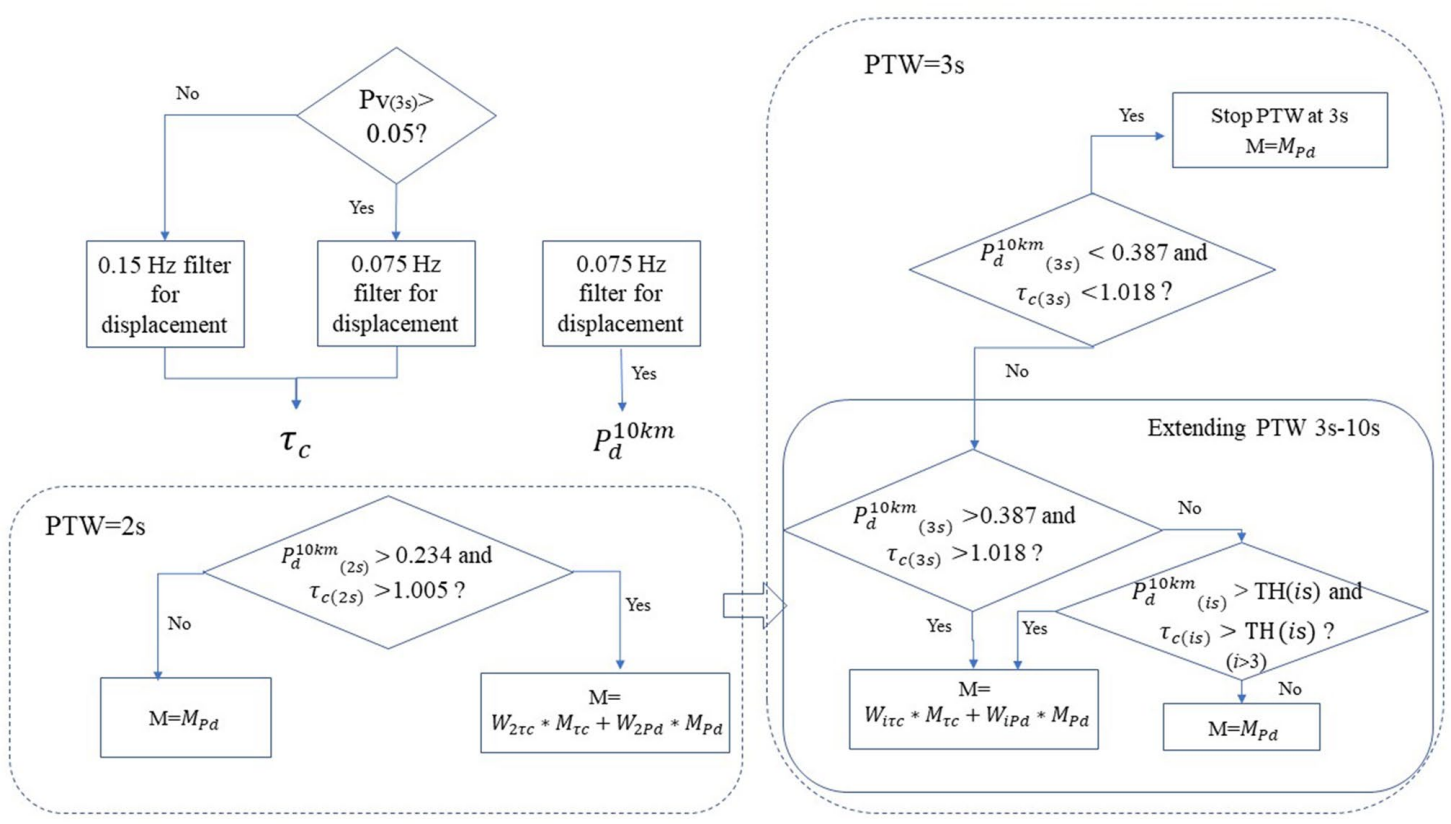
Process of the threshold-based evolutionary magnitude estimation algorithm. W_iτc_ and W_iPd_ are the weights of τ_c_ and P_d_^10km^, respectively, for PTW = *i* s. TH(*i*s) is the threshold of τ_c_ and P_d_^10km^ for PTW = *i* s.

For clarity, we have summarized the steps of our evolutionary magnitude estimation process in a flow chart (Fig. 6).

### Tests and analyses of robustness

To further investigate the reliability of the established relationships and our threshold-based approach, we applied our method to the validation dataset to evaluate the magnitude estimates, and we demonstrated the evolutionary magnitude estimation algorithm for the largest earthquake considered herein: the Ms 8.0 Wenchuan earthquake.

**Figure 7:**
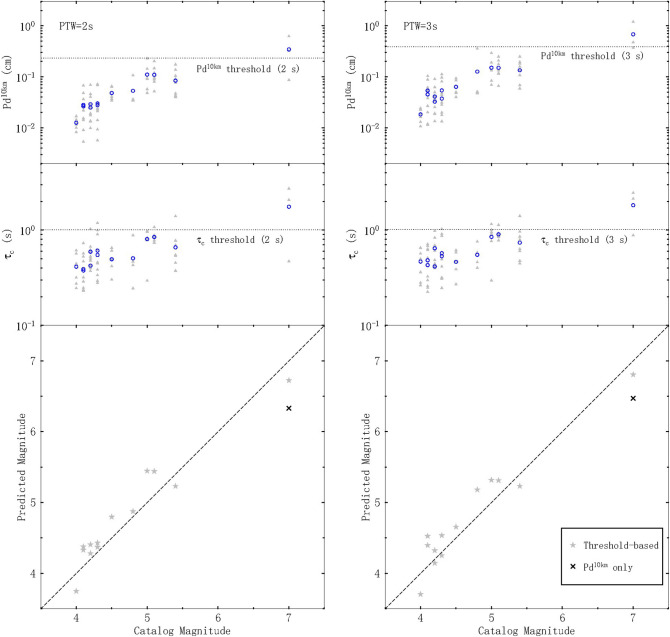
Magnitude estimation performance using the established relationships for 2016–2017 earthquakes. Gray triangles and blue circles indicate the parameter values for each record and the average values, respectively. Dashed lines and gray stars denote the 1:1 relationship and predicted magnitudes, respectively, using our threshold-based approach. The cross in each panel indicates the estimate for the M = 7 earthquake obtained using P_d_^10km^ only.

Figure 7 illustrates our magnitude estimation results for the 13 earthquakes that occurred during 2016–2017. We computed both P_d_^10km^ and τ_c_ only when data were available for PTW = 2 s. After comparisons with the corresponding thresholds (dashed lines), magnitude estimation was undertaken by following the threshold-based magnitude estimation steps presented in Fig. 6. We compared the average predicted magnitude with the catalog magnitude and found that P_d_ can provide robust magnitude estimates with a low standard deviation for all small events. In addition, the inclusion of τ_c_ allows the magnitude for large events to be more precisely estimated than using P_d_ alone: our method produced estimates of 6.72 and 6.33 for the M = 7 Jiuzhaigou earthquake with PTW = 2 s for the estimation with and without τ_c_, respectively. These results indicate that our method is stable and that it performed well for all 13 earthquakes considered with an average magnitude estimation error of 0.22 magnitude units for both PTW = 2 s and PTW = 3 s.

**Figure 8:**
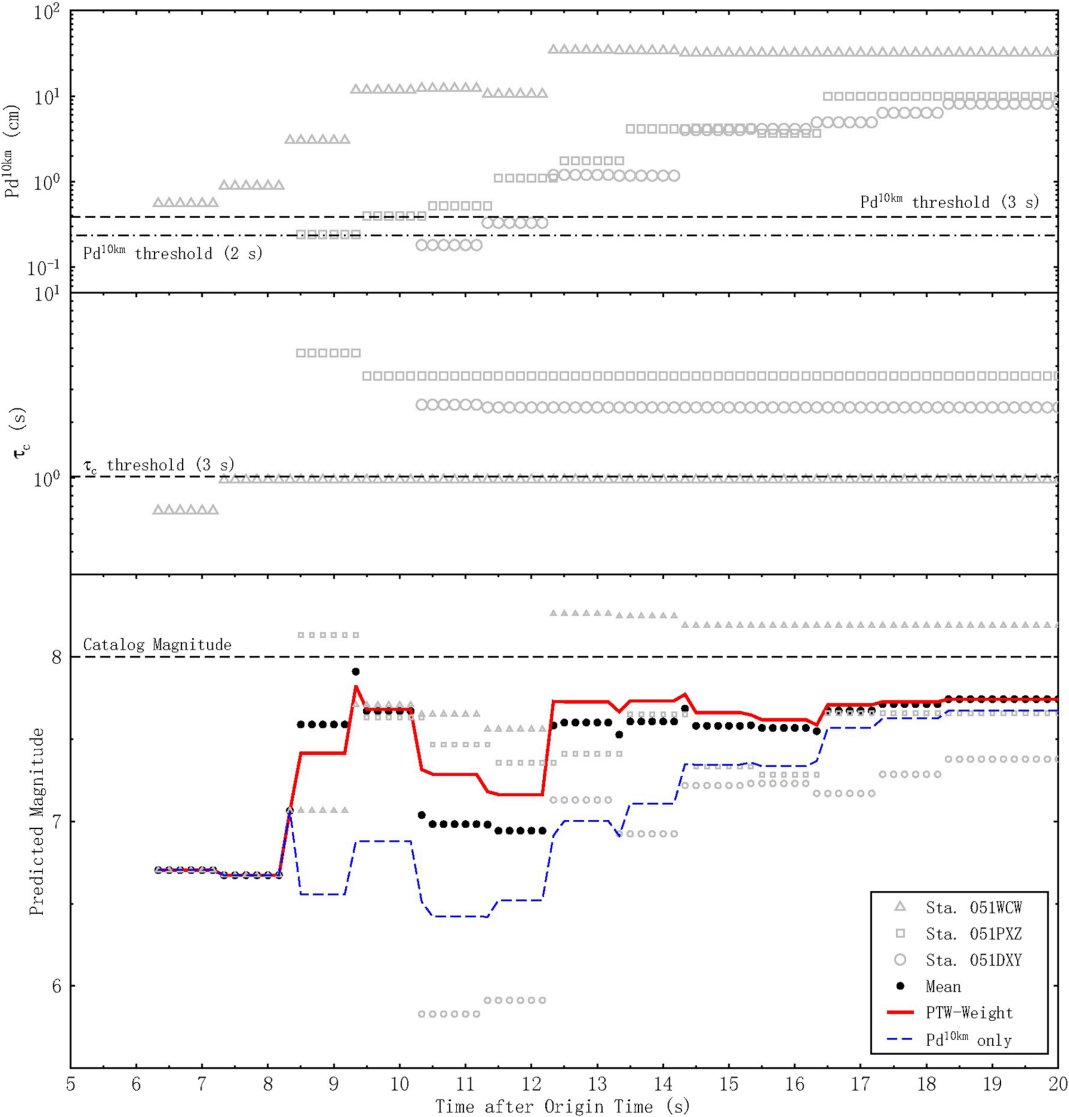
Temporal evolution of the magnitude estimates for the Ms 8.0 Wenchuan earthquake. Gray triangles, squares and circles represent stations 051WCW, 051PXZ and 051DXY, respectively. Black circles denote directly averaged estimates for the three stations. The dashed blue line indicates the estimates obtained using P_d_^10km^ only. The red line indicates the final averaged magnitude estimates based on the length of the PTW.

Figure 8 illustrates the evolutionary magnitude estimation results for the Wenchuan earthquake. Nearly 6.2 s after the origin time (OT), the first triggered station (051WCW) received 2 s of data after the P-wave arrival. Accordingly, because the τ_c_ value did not reach the corresponding threshold, only P_d_^10km^ was used, and the proposed method yielded a magnitude estimate of 6.7. Approximately 8.5 s after the OT, both P_d_^10km^ and τ_c_ exceeded their respective thresholds at the second triggered station (051PXZ), yielding a magnitude estimate of 8.13 at this station when PTW = 2 s. After averaging the estimates from the first two stations, a magnitude estimate of 7.41 was obtained just 8.5 s after the OT, reflecting a smaller underestimation error of 0.59 compared to the result (1.47) of using P_d_^10km^ only (dashed blue line). The third station (051PXZ) exhibited small displacement amplitudes at 2 and 3 s; these displacements were insufficient to reach P_d_^10km^ threshold. Accordingly, the estimates using P_d_^10km^ only for this station lowered the average magnitude for the three stations 10.3–12.3 s after the OT. We included the average estimates without considering the PTW length for all three stations (black circles) in Fig. 8 for a comparison with our averaged values based on the length of the PTW (red line). By averaging the estimates for the stations based on the PTW, the magnitude estimation error was reduced from 1 to 0.7 magnitude units when the third station had a short PTW (2 s). With increasing PTW, the final estimates converged to 7.74 at 12.3 s after the OT.

## Discussion and conclusions

We investigated two earthquake magnitude estimation parameters (τ_c_ and P_d_) based on CSMNC data during 2007–2015 for the Sichuan–Yunnan region. In particular, we established a τ_c_-magnitude relationship with a fixed PTW of 3 s and a P_d_^10km^-magnitude relationship with increasing PTW (2–10 s). Based on the derived relationships, we proposed a threshold-based evolutionary approach for estimating the event magnitude within seconds of the P-wave arrival.

Our study highlights the shortcomings of existing methods, for which the applications of P_d_ and τ_c_ are limited by the length of the PTW and uncertainty, respectively. Although P_d_ produces a consistent, small error for small-moderate earthquakes, the complex moment rate function and incomplete rupture typical of large events preclude a simple prediction of the final magnitude using P_d_ in the first few seconds of an event. The PTW must be extended sufficiently to capture the information released by large earthquakes. Conversely, τ_c_ exhibits better performance in predicting high event magnitudes for short PTWs. However, we observed large scatter of τ_c_ in the low-magnitude range, which is consistent with the results of Zollo et al.^16^. Since τ_c_ is more sensitive than P_d_ to the low-frequency drift caused by integration, we retained the 0.075 Hz high-pass filter for P_d_ and applied a criterion that allows a filter with a higher cutoff frequency to be selected for τ_c_ based on the P_v_ threshold (0.05 cm/s); this selective criterion reduces such errors considerably.

Our proposed threshold-based methodology is based primarily on P_d_ results and setting a threshold to ensure that τ_c_ participates in the magnitude estimation process only for large earthquakes. Previous studies have proposed using τ_c_ and P_d_ together to improve reliability, but the occurrence of abnormally high τ_c_ values for small events will introduce errors when combining these two parameters. Our approach applies an advance judgment (compared with a predefined threshold) and implements reasonable strategies based on the levels of the observed parameters. Specifically, situation (1) indicates that an event is most likely a large event, so we jointly use the estimates of τ_c_ and P_d_^10km^. Situation (2) indicates that an event may be a large earthquake with a small P_d_^10km^ or a small-moderate earthquake with an unexpectedly large τ_c_. To avoid the potentially serious consequences of failing to trigger an alarm for a large earthquake, we suggest extending the PTW and using only P_d_^10km^ for the magnitude estimation in this scenario. Although a small P_d_^10km^ will underestimate a potential large event, this underestimation can be improved by extending PTW, which helps mitigate the magnitude overestimation for potential small-moderate earthquakes caused by combining unexpectedly large τ_c_ values. Situation (3) indicates that an event may be a large earthquake with an unexpectedly small τ_c_ or a small-moderate earthquake with a large P_d_^10km^. Considering the small error of P_d_^10km^ found in the small-moderate magnitude range and the underestimation of potential large earthquakes caused by jointly using small τ_c_ values, we also suggest extending the PTW to avoid excluding large earthquakes and using only P_d_^10km^ for the magnitude estimation in this case. Situation (4) indicates that an event is most likely a small-moderate event, so we use the estimates of only P_d_^10km^ and cease extending PTW beyond 3 s. Accordingly, this approach integrates the timeliness of estimating large events based on τ_c_ and the robustness of general magnitude estimation based on P_d_; moreover, the proposed method reduces the impacts of the uncertainty in τ_c_ and the magnitude saturation of P_d_. We believe this approach provides the most accurate estimate in the shortest time.

Offline applications to 13 events that occurred during 2016–2017 and the Ms 8.0 Wenchuan earthquake demonstrated the robustness and feasibility of our proposed methodology. According to the simulated results, the average magnitude estimation error of 0.2 magnitude units can be achieved using a PTW of 2 s for earthquakes with M ≤ 7. For the largest event considered herein, namely, the Ms 8.0 Wenchuan earthquake, a relatively stable magnitude estimate of 7.74 was obtained 12.3 s after the OT. Our approach offers clear advantages over the prediction approach using P_d_^10km^ alone. Moreover, we show that the pull-down effects of distant stations with short PTWs can be mitigated by averaging the magnitude estimates based on the lengths of the records available at those stations, thereby reducing the weights of stations with short PTWs. Since the underestimation caused by the third station (051PXZ) was so obvious, for future real-time application, we suggest retaining the previous magnitude estimation results without introducing low-amplitude stations if the event is judged most likely to be a large earthquake based on the near-source stations. It is important to note that the distances used in this study were based on the post-location. Accordingly, the real performance will be affected by the error in locating earthquakes in real-time.

In addition to robustly declaring the event magnitude, it is equally important for an EEW system to send an alert for the largest lead time (i.e., the time interval between the alarm being raised and the arrival of destructive S waves at the target site). With regard to the timeliness, the proposed method needs only to obtain the initial information after P-wave arrivals, following which the method can automatically choose a specific strategy based on pre-established thresholds and magnitude relationships without complex computations; hence, this magnitude estimation method requires little execution time. Typically, the lead time is controlled by the difference in the P-S-wave traveltimes. Assuming a mean station spacing of 20 km within a network ensures that 3 stations are triggered within 5 s of the OT^10^, and our approach will provide a robust magnitude estimate for M < 6.5 earthquakes within 8 s after the OT. Therefore, assuming Vs = 3.5 km/s, the lead time corresponding to a specific hypocentral distance (R) between the epicenter and target will be given by (R/3.5–8) s. A lead time of 6 s is available for a hypocentral distance of 50 km, and 20 s is available at 100 km. Specifically, for the Ms 8.0 Wenchuan earthquake, a relatively stable magnitude estimate of 7.74 can be obtained 12.3 s after the OT by our approach. Note that the region that was severely damaged by the Wenchuan earthquake (Beichuan) was almost 90 km away from the epicenter^31^; our method would provide the people in Beichuan a lead time of 13.4 s. Indeed, expanding the PTW of near-source stations cannot avoid the inclusion of S-waves; however, stable magnitude estimates were provided by our approach without severe overestimation caused by the contamination of S waves.

The proposed method was conceived to be applied to a regional EEW system and to provide robust source parameters (magnitude) with continuous information from a dense network. Our approach begins to provide a magnitude estimate after the first station is triggered, similar to the single-station, threshold-based onsite approach, which quickly provides approximate warnings on whether the following ground motion will be damaged. Similarly, our approach uses a four-entry decision table composed of τ_c_ and P_d_^10km^ thresholds to increase the reliability of the preliminary determination on whether the event is large, and based on the judgment for selectively applying period and amplitude parameters to predict the magnitude at the early stage of earthquake occurrence. When continuous waveform data from multiple stations are streamed to the network processing center, our approach combines the information from all available stations and chooses whether to extend the PTW to update the source parameters to provide the most robust warning, similar to a regional (network-based) warning. We believe this method can be widely used in other geographical regions to improve the robustness and timeliness of magnitude estimation. Accurate magnitude estimations closely related to the determination of a potential damage area will help those areas that will not receive alarms due to an underestimated magnitude; this will be conducive to reducing earthquake hazards and providing guiding to the emergency response a few seconds in advance.

## Methods

An automatic P-wave picker described by Allen^32^ was used to detect P-waves from vertical acceleration records, and the waveforms were checked manually to correct the P-wave arrivals as necessary. The acceleration signals were integrated once (twice) with respect to the velocity (displacement). A four-pole 0.075 Hz high-pass filter was used to remove low-frequency drift in the velocity and displacement. The peak amplitude P_d_ is the maximum absolute value of the vertical displacement in the first several seconds after the P-wave arrival. We applied an extended P-wave time window (2–10 s) to obtain the coefficients for the P_d_ attenuation relationship through least-squares multi-regression analysis:

7 $$\log \left( {P_{d} } \right) = A * M + B * log\left( R \right) + C$$

where P_d_, M and R are the peak displacement (cm), magnitude and hypocentral distance (km), respectively.

To retrieve the magnitude dependence of P_d_, we normalized P_d_ to a reference distance of 10 km to obtain P_d_^10km^ and calibrated the linear regression relationship between the event-averaged P_d_^10km^ and magnitude.

The parameter τ_c_ is defined as follows.

8 $$\tau_{c} = {{2\pi } \mathord{\left/ {\vphantom {{2\pi } {\sqrt r }}} \right. \kern-\nulldelimiterspace} {\sqrt r }} = {{2\pi } \mathord{\left/ {\vphantom {{2\pi } {\sqrt {{{\int_{0}^{{\tau_{0} }} {v\left( t \right)dt} } \mathord{\left/ {\vphantom {{\int_{0}^{{\tau_{0} }} {v\left( t \right)dt} } {\int_{0}^{{\tau_{0} }} {u\left( t \right)dt} }}} \right. \kern-\nulldelimiterspace} {\int_{0}^{{\tau_{0} }} {u\left( t \right)dt} }}} }}} \right. \kern-\nulldelimiterspace} {\sqrt {{{\int_{0}^{{\tau_{0} }} {v^2\left( t \right)dt} } \mathord{\left/ {\vphantom {{\int_{0}^{{\tau_{0} }} {v\left( t \right)dt} } {\int_{0}^{{\tau_{0} }} {u\left( t \right)dt} }}} \right. \kern-\nulldelimiterspace} {\int_{0}^{{\tau_{0} }} {u^2\left( t \right)dt} }}} }}$$

where u(t) and v(t) are the vertical displacement and velocity, respectively. Here, τ_0_ is the time window over which τ_c_ is computed, starting at the first P-wave arrival and typically equaling 3 s. We averaged the τ_c_ values for all records for each event and used the least-squares method to fit an event-averaged τ_c_ value; this reduced scatter induced by site conditions and radiation patterns.

## Supplementary information

Supplementary Information 1.

Supplementary Information 2.

## Data Availability

All data generated or analyzed during this study are available in the Supplementary Information files.
