# Supplementary material for: Threshold-based evolutionary magnitude estimation for an earthquake early warning system in the Sichuan–Yunnan region, China
Source: Sci Rep. 2020 Dec 3;10:21055. doi: 10.1038/s41598-020-78046-2 (PMC7713221; doi:10.1038/s41598-020-78046-2)
Supplement: Supplementary file 1 — Supplementary Information 1. [file 41598_2020_78046_MOESM1_ESM.docx]

Electronic Supplementary Material to

**Threshold-based evolutionary magnitude estimation for an earthquake early warning system in the Sichuan–Yunnan region, China**

By Yuan Wang ^a^, Shanyou Li ^a^, Jindong Song ^a^

^a^ Institute of Engineering Mechanics, China Earthquake Administration; Key Laboratory of Earthquake Engineering and Engineering Vibration of China Earthquake Administration, Harbin 150080, China

The supplementary materials show thresholds of τ_c_ and P_d_^10km^ corresponding to magnitude of 6.5 for PTW = 3 s (Figure S1), list of events used in this study (Table S1), coefficients for P_d_^10km^ magnitude scaling relations (Table S2), thresholds for M = 6.5 (Table S3) and weights for magnitude estimation (Table S4). All figures and tables are in the Microsoft word files.

**Supplementary Material**


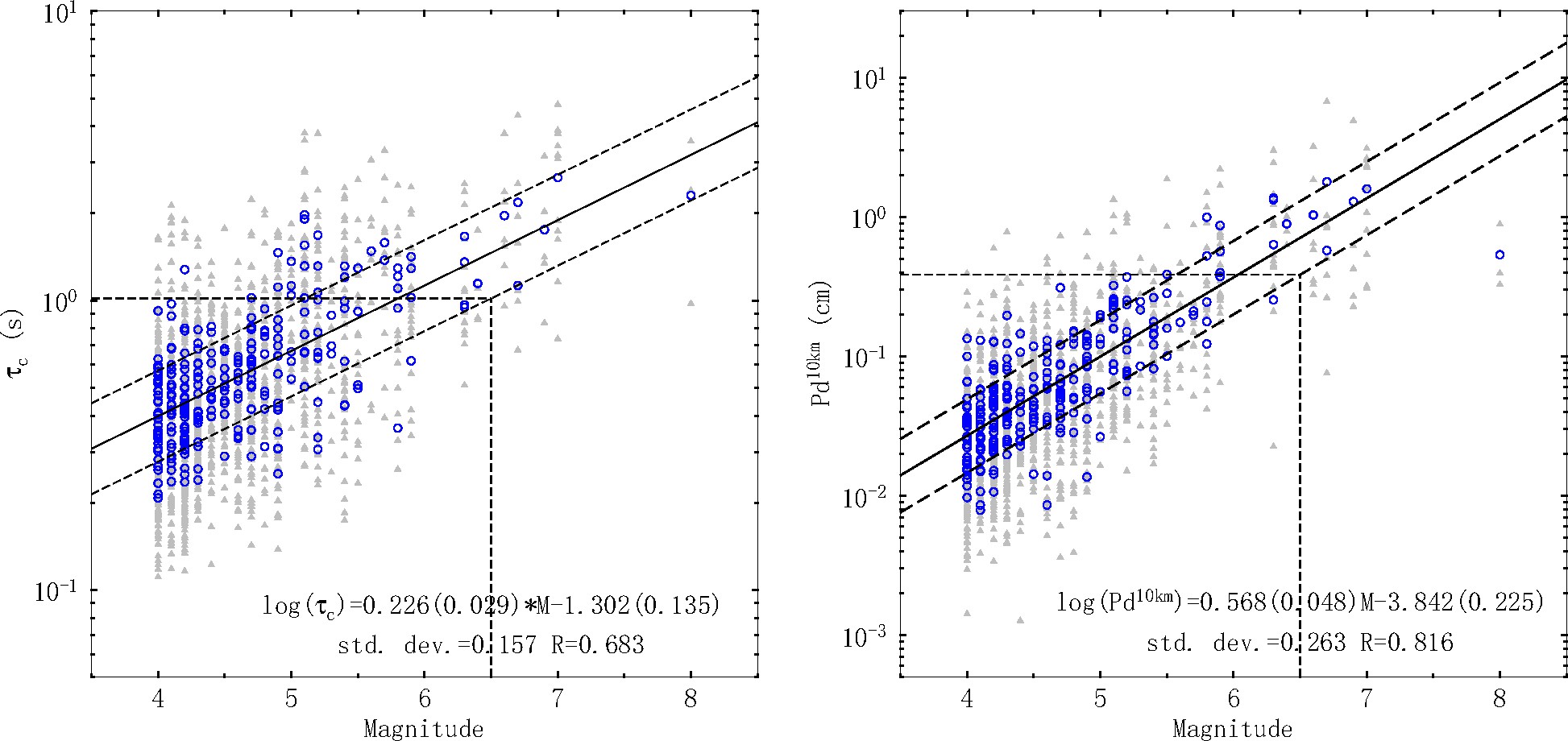


Figure S1. Thresholds of τ_c_ and P_d_^10km^ corresponding to magnitude of 6.5 for PTW = 3 s. Individual records denoted by gray triangles and averaged values by blue circles. Solid line indicates fitting relationship. Misfit standard error bounds (dashed lines) represent expected ±1σ variation.

**Table S1**

**List of events from fitting dataset in the period of 2007-2015**

| Event date | Time | Long. | Lat. | Depth(km) | Ms | Num. of records |
| --- | --- | --- | --- | --- | --- | --- |
| 2007/6/3 | 5:34:56 | 101.1 | 23.1 | 6 | 6.7 | 5 |
| 2007/6/3 | 8:09:37 | 101.2 | 22.9 | 15 | 4.1 | 3 |
| 2007/6/3 | 10:49:01 | 101.2 | 23.0 | 11 | 4.9 | 5 |
| 2007/7/10 | 8:03:47 | 74.5 | 39.8 | 45 | 4.2 | 4 |
| 2007/7/25 | 18:06:11 | 77.2 | 39.7 | 38 | 4.2 | 6 |
| 2007/7/27 | 13:18:11 | 77.3 | 39.6 | 18 | 4.3 | 5 |
| 2007/11/28 | 18:38:30 | 81.8 | 41.7 | 28 | 4.2 | 3 |
| 2007/12/21 | 8:45:03 | 77.0 | 39.7 | 31 | 4.4 | 5 |
| 2008/2/18 | 10:44:46 | 99.9 | 25.8 | 14 | 4.6 | 5 |
| 2008/3/30 | 16:32:25 | 101.9 | 38.0 | 11 | 5.2 | 6 |
| 2008/4/10 | 15:17:21 | 75.1 | 39.6 | 13 | 4.8 | 3 |
| 2008/4/11 | 4:25:47 | 82.4 | 41.5 | 15 | 4.0 | 3 |
| 2008/5/12 | 14:28:04 | 103.4 | 31.0 | 14 | 8.0 | 3 |
| 2008/5/12 | 14:43:15 | 103.8 | 31.3 | 14 | 6.3 | 4 |
| 2008/5/12 | 14:54:17 | 103.6 | 31.3 | 13 | 5.8 | 4 |
| 2008/5/12 | 15:34:42 | 103.8 | 31.3 | 13 | 5.8 | 3 |
| 2008/5/12 | 15:44:16 | 103.9 | 31.3 | 9 | 4.6 | 3 |
| 2008/5/12 | 15:45:33 | 103.8 | 31.3 | 10 | 4.7 | 4 |
| 2008/5/12 | 16:02:58 | 104.4 | 31.8 | 9 | 4.7 | 3 |
| 2008/5/12 | 16:10:57 | 103.6 | 31.1 | 10 | 5.5 | 3 |
| 2008/5/12 | 16:21:40 | 104.3 | 31.5 | 11 | 5.5 | 6 |
| 2008/5/12 | 16:26:12 | 104.1 | 31.4 | 12 | 5.1 | 4 |
| 2008/5/12 | 16:40:30 | 103.5 | 31.4 | 12 | 4.2 | 4 |
| 2008/5/12 | 17:06:59 | 103.7 | 31.2 | 10 | 5.2 | 3 |
| 2008/5/12 | 17:23:35 | 104.9 | 32.2 | 20 | 5.0 | 3 |
| 2008/5/12 | 17:31:15 | 103.6 | 31.2 | 10 | 5.2 | 4 |
| 2008/5/12 | 17:42:24 | 104.1 | 31.5 | 14 | 5.3 | 4 |
| 2008/5/12 | 18:19:15 | 103.6 | 31.3 | 10 | 4.0 | 4 |
| 2008/5/12 | 18:43:12 | 104.1 | 31.5 | 14 | 4.6 | 4 |
| 2008/5/12 | 19:11:01 | 103.7 | 31.3 | 14 | 6.3 | 5 |
| 2008/5/12 | 19:25:55 | 103.5 | 31.3 | 9 | 4.0 | 3 |
| 2008/5/12 | 19:41:09 | 105.1 | 32.2 | 9 | 4.8 | 3 |
| 2008/5/12 | 20:13:48 | 103.6 | 31.4 | 20 | 4.3 | 3 |
| 2008/5/12 | 20:15:40 | 104.6 | 31.9 | 9 | 4.9 | 3 |
| 2008/5/12 | 22:09:48 | 104.4 | 32.0 | 10 | 4.6 | 3 |
| 2008/5/12 | 22:10:24 | 103.6 | 31.3 | 18 | 4.6 | 3 |
| 2008/5/12 | 22:15:27 | 104.8 | 32.1 | 19 | 4.6 | 3 |
| 2008/5/12 | 23:05:30 | 103.8 | 31.2 | 17 | 5.2 | 3 |
| 2008/5/13 | 0:28:53 | 103.8 | 31.3 | 16 | 4.5 | 3 |
| 2008/5/13 | 1:29:06 | 103.7 | 31.2 | 24 | 4.9 | 3 |
| 2008/5/13 | 1:52:24 | 104.6 | 31.8 | 30 | 4.7 | 3 |
| 2008/5/13 | 1:54:32 | 103.6 | 31.3 | 17 | 5.1 | 3 |
| 2008/5/13 | 4:08:49 | 104.1 | 31.4 | 21 | 5.8 | 3 |
| 2008/5/13 | 4:45:31 | 104.6 | 31.7 | 20 | 5.2 | 5 |
| 2008/5/13 | 4:48:55 | 103.6 | 31.2 | 10 | 4.1 | 3 |
| 2008/5/13 | 6:19:30 | 104.3 | 31.8 | 10 | 4.1 | 3 |
| 2008/5/13 | 6:24:41 | 105.1 | 32.2 | 15 | 4.2 | 4 |
| 2008/5/13 | 6:47:21 | 103.7 | 31.2 | 9 | 4.3 | 3 |
| 2008/5/13 | 7:46:18 | 103.6 | 31.3 | 13 | 5.4 | 3 |
| 2008/5/13 | 7:54:46 | 103.6 | 31.3 | 10 | 5.2 | 3 |
| 2008/5/13 | 10:15:16 | 104.1 | 31.6 | 15 | 4.3 | 3 |
| 2008/5/13 | 11:00:38 | 103.7 | 31.2 | 14 | 4.9 | 3 |
| 2008/5/13 | 11:09:54 | 103.6 | 31.2 | 10 | 4.0 | 3 |
| 2008/5/13 | 14:39:51 | 104.3 | 31.6 | 23 | 4.2 | 3 |
| 2008/5/13 | 17:41:21 | 104.5 | 32.0 | 20 | 4.2 | 4 |
| 2008/5/13 | 18:16:06 | 104.5 | 31.7 | 12 | 4.8 | 5 |
| 2008/5/13 | 21:24:04 | 104.0 | 31.5 | 10 | 4.0 | 3 |
| 2008/5/14 | 10:54:37 | 103.6 | 31.3 | 16 | 5.8 | 3 |
| 2008/5/14 | 13:54:57 | 104.2 | 32.0 | 15 | 4.7 | 3 |
| 2008/5/14 | 17:26:43 | 104.1 | 31.4 | 10 | 5.1 | 3 |
| 2008/5/15 | 1:33:24 | 103.7 | 31.3 | 15 | 4.7 | 3 |
| 2008/5/16 | 6:47:42 | 103.6 | 31.4 | 17 | 4.0 | 3 |
| 2008/5/16 | 13:25:47 | 103.4 | 31.3 | 14 | 5.9 | 5 |
| 2008/5/16 | 13:53:06 | 103.6 | 31.2 | 10 | 4.0 | 3 |
| 2008/6/18 | 20:59:33 | 102.4 | 29.2 | 7 | 4.4 | 5 |
| 2008/8/30 | 16:30:53 | 102.1 | 26.3 | 19 | 6.3 | 3 |
| 2008/12/26 | 2:19:55 | 103.0 | 24.9 | 11 | 4.3 | 33 |
| 2009/2/17 | 12:42:51 | 75.6 | 39.9 | 13 | 4.0 | 3 |
| 2009/3/28 | 19:11:21 | 112.9 | 38.9 | 8 | 4.3 | 3 |
| 2009/4/14 | 4:37:10 | 99.8 | 26.0 | 10 | 4.6 | 7 |
| 2009/4/22 | 17:26:03 | 77.3 | 40.1 | 25 | 5.0 | 3 |
| 2009/6/30 | 2:03:52 | 104.0 | 31.5 | 24 | 5.5 | 4 |
| 2009/6/30 | 13:40:25 | 104.0 | 31.5 | 10 | 4.2 | 3 |
| 2009/6/30 | 15:22:21 | 104.0 | 31.5 | 24 | 5.0 | 3 |
| 2009/7/9 | 19:19:14 | 101.0 | 25.6 | 6 | 6.3 | 3 |
| 2009/7/10 | 17:02:01 | 101.1 | 25.6 | 10 | 5.4 | 3 |
| 2009/7/10 | 20:57:31 | 101.0 | 25.6 | 13 | 4.7 | 3 |
| 2009/9/2 | 18:16:10 | 81.5 | 41.7 | 10 | 4.5 | 3 |
| 2009/11/2 | 5:07:16 | 100.7 | 25.9 | 10 | 5.0 | 6 |
| 2009/11/5 | 7:31:33 | 109.1 | 34.5 | 5 | 4.2 | 8 |
| 2009/11/21 | 15:51:01 | 106.6 | 38.2 | 9 | 4.3 | 20 |
| 2009/12/11 | 3:23:12 | 100.9 | 27.1 | 4 | 4.0 | 3 |
| 2010/1/1 | 10:08:20 | 99.8 | 26.3 | 11 | 4.6 | 6 |
| 2010/1/24 | 10:36:11 | 110.8 | 35.6 | 8 | 4.8 | 6 |
| 2010/2/17 | 8:39:03 | 74.8 | 39.5 | 7 | 4.2 | 3 |
| 2010/4/3 | 4:26:31 | 100.2 | 22.4 | 10 | 4.1 | 3 |
| 2010/4/9 | 18:51:58 | 118.1 | 39.5 | 13 | 4.1 | 14 |
| 2010/4/15 | 8:55:52 | 76.6 | 40.1 | 15 | 4.3 | 4 |
| 2010/6/1 | 23:58:08 | 99.2 | 24.9 | 5 | 4.9 | 4 |
| 2010/6/5 | 20:58:11 | 112.6 | 38.2 | 6 | 4.6 | 6 |
| 2010/6/10 | 14:38:02 | 74.7 | 39.9 | 9 | 4.9 | 3 |
| 2010/6/22 | 16:46:01 | 106.2 | 38.3 | 6 | 4.4 | 18 |
| 2010/8/29 | 8:53:27 | 103.0 | 27.1 | 10 | 4.9 | 4 |
| 2010/10/18 | 5:49:38 | 104.1 | 28.1 | 11 | 4.7 | 3 |
| 2010/12/3 | 22:30:56 | 103.0 | 27.1 | 9 | 4.3 | 3 |
| 2011/1/1 | 9:56:05 | 75.2 | 39.5 | 9 | 4.9 | 9 |
| 2011/1/8 | 14:36:05 | 84.2 | 42.1 | 8 | 4.1 | 3 |
| 2011/1/16 | 8:30:16 | 104.5 | 32.0 | 15 | 4.1 | 5 |
| 2011/2/12 | 13:44:16 | 103.0 | 27.1 | 12 | 4.5 | 6 |
| 2011/2/23 | 21:32:12 | 103.8 | 34.3 | 8 | 4.2 | 3 |
| 2011/3/2 | 9:18:34 | 75.3 | 39.0 | 7 | 4.9 | 4 |
| 2011/4/1 | 13:07:12 | 104.0 | 31.6 | 10 | 4.0 | 4 |
| 2011/4/10 | 17:02:42 | 100.8 | 31.3 | 10 | 5.4 | 5 |
| 2011/4/15 | 15:44:46 | 102.9 | 26.7 | 12 | 4.5 | 5 |
| 2011/5/6 | 18:48:15 | 103.6 | 31.3 | 15 | 4.1 | 4 |
| 2011/5/15 | 15:05:32 | 105.3 | 32.6 | 17 | 4.8 | 3 |
| 2011/5/23 | 21:22:28 | 74.7 | 39.5 | 8 | 4.2 | 4 |
| 2011/5/31 | 21:13:37 | 98.7 | 25.0 | 11 | 4.5 | 4 |
| 2011/6/5 | 13:21:45 | 104.1 | 31.8 | 19 | 4.2 | 8 |
| 2011/8/2 | 19:57:14 | 112.0 | 36.5 | 19 | 4.1 | 3 |
| 2011/8/9 | 19:50:17 | 98.7 | 25.0 | 11 | 5.2 | 7 |
| 2011/8/11 | 18:06:29 | 77.2 | 39.9 | 10 | 5.8 | 4 |
| 2011/8/31 | 0:23:51 | 98.0 | 24.7 | 10 | 4.3 | 4 |
| 2011/9/4 | 12:13:45 | 103.6 | 31.3 | 21 | 4.2 | 6 |
| 2011/10/28 | 9:40:51 | 104.8 | 32.3 | 15 | 4.3 | 4 |
| 2011/11/1 | 5:58:15 | 105.3 | 32.6 | 6 | 5.2 | 5 |
| 2011/12/6 | 4:37:47 | 103.2 | 27.4 | 5 | 4.0 | 3 |
| 2011/12/12 | 7:51:26 | 77.3 | 39.6 | 10 | 4.1 | 3 |
| 2011/12/26 | 0:46:52 | 103.8 | 31.4 | 20 | 4.7 | 7 |
| 2012/1/3 | 15:39:57 | 77.3 | 39.9 | 9 | 4.0 | 3 |
| 2012/2/2 | 5:16:57 | 122.4 | 40.5 | 9 | 4.2 | 3 |
| 2012/3/5 | 1:11:46 | 101.8 | 30.1 | 5 | 4.1 | 3 |
| 2012/4/5 | 6:32:49 | 103.1 | 32.0 | 12 | 4.1 | 5 |
| 2012/4/13 | 14:47:59 | 75.2 | 39.9 | 7 | 4.0 | 4 |
| 2012/4/18 | 22:47:59 | 77.2 | 39.9 | 7 | 4.2 | 3 |
| 2012/4/18 | 22:49:34 | 77.2 | 40.0 | 7 | 4.1 | 3 |
| 2012/5/11 | 18:18:09 | 102.0 | 37.8 | 16 | 4.9 | 10 |
| 2012/5/27 | 19:19:01 | 103.9 | 31.5 | 20 | 4.0 | 5 |
| 2012/5/28 | 10:22:52 | 118.5 | 39.7 | 22 | 4.7 | 7 |
| 2012/6/11 | 23:53:58 | 103.5 | 31.3 | 20 | 4.4 | 4 |
| 2012/6/15 | 5:51:26 | 84.2 | 42.2 | 10 | 5.3 | 3 |
| 2012/6/18 | 3:05:13 | 117.6 | 39.6 | 5 | 4.0 | 38 |
| 2012/6/24 | 15:59:34 | 100.7 | 27.7 | 11 | 5.7 | 3 |
| 2012/7/2 | 6:17:59 | 103.5 | 31.7 | 18 | 4.0 | 7 |
| 2012/7/18 | 4:18:42 | 102.3 | 29.0 | 13 | 4.2 | 4 |
| 2012/7/20 | 20:11:51 | 119.6 | 33.0 | 15 | 4.9 | 3 |
| 2012/7/30 | 0:05:33 | 101.2 | 23.1 | 10 | 4.2 | 8 |
| 2012/8/1 | 17:52:20 | 75.5 | 39.7 | 32 | 4.8 | 9 |
| 2012/8/7 | 17:43:25 | 77.4 | 39.5 | 12 | 4.4 | 6 |
| 2012/8/26 | 7:13:34 | 117.4 | 39.6 | 9 | 4.0 | 6 |
| 2012/9/7 | 11:19:42 | 104.0 | 27.5 | 14 | 5.7 | 10 |
| 2012/9/7 | 11:58:01 | 104.0 | 27.6 | 9 | 4.4 | 5 |
| 2012/9/7 | 12:16:30 | 104.0 | 27.6 | 14 | 5.6 | 9 |
| 2012/9/7 | 13:12:46 | 104.0 | 27.5 | 14 | 4.4 | 4 |
| 2012/10/8 | 23:05:58 | 103.9 | 31.4 | 25 | 4.2 | 5 |
| 2012/10/19 | 10:44:17 | 102.9 | 30.1 | 26 | 4.1 | 13 |
| 2012/11/14 | 19:42:56 | 102.5 | 26.5 | 13 | 4.2 | 4 |
| 2012/11/20 | 10:24:25 | 106.3 | 38.4 | 21 | 4.6 | 21 |
| 2012/12/1 | 9:59:04 | 75.6 | 39.2 | 6 | 4.0 | 3 |
| 2012/12/1 | 23:16:44 | 104.2 | 31.9 | 24 | 4.3 | 9 |
| 2013/1/5 | 13:06:13 | 104.1 | 31.7 | 23 | 4.4 | 7 |
| 2013/1/23 | 12:18:15 | 123.2 | 41.5 | 10 | 5.1 | 3 |
| 2013/1/26 | 23:41:14 | 77.4 | 39.9 | 8 | 4.1 | 3 |
| 2013/1/27 | 13:06:47 | 103.3 | 31.5 | 16 | 4.2 | 7 |
| 2013/2/5 | 21:13:50 | 100.1 | 39.0 | 15 | 4.0 | 4 |
| 2013/2/18 | 14:44:19 | 105.0 | 32.3 | 24 | 4.0 | 3 |
| 2013/2/19 | 10:47:00 | 103.1 | 27.1 | 10 | 4.9 | 13 |
| 2013/2/20 | 13:01:57 | 101.6 | 23.3 | 15 | 4.8 | 3 |
| 2013/2/22 | 5:43:41 | 100.8 | 26.7 | 14 | 4.2 | 7 |
| 2013/3/3 | 13:41:16 | 99.7 | 25.9 | 9 | 5.5 | 8 |
| 2013/3/9 | 20:23:58 | 103.5 | 31.4 | 20 | 4.2 | 7 |
| 2013/3/11 | 11:01:37 | 77.5 | 40.2 | 10 | 5.1 | 4 |
| 2013/3/19 | 9:47:15 | 101.0 | 23.1 | 10 | 4.1 | 4 |
| 2013/4/17 | 9:45:54 | 99.8 | 25.9 | 11 | 5.1 | 15 |
| 2013/4/18 | 11:46:01 | 99.8 | 25.9 | 9 | 4.1 | 6 |
| 2013/4/20 | 8:02:46 | 103.0 | 30.3 | 13 | 7.0 | 11 |
| 2013/4/20 | 8:06:35 | 102.9 | 30.2 | 10 | 4.7 | 4 |
| 2013/4/20 | 8:07:30 | 102.9 | 30.3 | 10 | 5.4 | 4 |
| 2013/4/20 | 8:31:38 | 103.0 | 30.4 | 12 | 4.5 | 5 |
| 2013/4/20 | 8:37:14 | 102.9 | 30.2 | 19 | 4.5 | 7 |
| 2013/4/20 | 8:42:35 | 102.9 | 30.3 | 22 | 4.2 | 5 |
| 2013/4/20 | 8:52:22 | 102.9 | 30.2 | 16 | 4.1 | 4 |
| 2013/4/20 | 9:02:58 | 102.9 | 30.3 | 15 | 4.8 | 6 |
| 2013/4/20 | 9:11:52 | 102.8 | 30.3 | 16 | 4.7 | 9 |
| 2013/4/20 | 9:20:11 | 103.0 | 30.2 | 19 | 4.8 | 10 |
| 2013/4/20 | 9:26:01 | 102.9 | 30.2 | 17 | 4.6 | 8 |
| 2013/4/20 | 9:37:29 | 103.0 | 30.3 | 15 | 4.9 | 10 |
| 2013/4/20 | 9:39:35 | 102.8 | 30.1 | 17 | 4.7 | 10 |
| 2013/4/20 | 10:01:41 | 102.9 | 30.2 | 15 | 4.1 | 3 |
| 2013/4/20 | 10:12:34 | 102.9 | 30.3 | 19 | 4.0 | 7 |
| 2013/4/20 | 10:19:05 | 102.9 | 30.3 | 15 | 4.3 | 6 |
| 2013/4/20 | 10:29:48 | 102.8 | 30.2 | 12 | 4.1 | 6 |
| 2013/4/20 | 10:38:37 | 103.0 | 30.3 | 17 | 4.7 | 11 |
| 2013/4/20 | 11:34:17 | 102.9 | 30.2 | 15 | 5.4 | 12 |
| 2013/4/20 | 15:18:34 | 103.0 | 30.4 | 17 | 4.2 | 4 |
| 2013/4/20 | 15:53:27 | 102.9 | 30.3 | 24 | 4.0 | 3 |
| 2013/4/20 | 17:45:14 | 103.0 | 30.3 | 17 | 4.0 | 10 |
| 2013/4/20 | 18:59:02 | 103.0 | 30.4 | 17 | 4.1 | 5 |
| 2013/4/20 | 19:12:50 | 103.0 | 30.4 | 17 | 4.3 | 11 |
| 2013/4/20 | 22:29:34 | 102.9 | 30.3 | 21 | 4.1 | 3 |
| 2013/4/21 | 0:14:36 | 102.8 | 30.1 | 10 | 4.0 | 11 |
| 2013/4/21 | 3:54:46 | 103.0 | 30.2 | 15 | 4.0 | 9 |
| 2013/4/21 | 4:53:44 | 103.1 | 30.4 | 27 | 5.4 | 13 |
| 2013/4/21 | 11:59:39 | 103.0 | 30.3 | 17 | 4.9 | 11 |
| 2013/4/21 | 12:39:18 | 102.9 | 30.2 | 23 | 4.2 | 4 |
| 2013/4/21 | 17:05:24 | 103.0 | 30.3 | 17 | 5.4 | 13 |
| 2013/4/21 | 18:48:24 | 103.0 | 30.3 | 18 | 4.2 | 11 |
| 2013/4/21 | 22:16:56 | 102.9 | 30.3 | 18 | 4.4 | 11 |
| 2013/4/21 | 23:25:23 | 102.8 | 30.3 | 26 | 4.0 | 6 |
| 2013/4/22 | 3:36:37 | 102.9 | 30.2 | 20 | 4.0 | 6 |
| 2013/4/23 | 5:54:50 | 103.0 | 30.4 | 19 | 4.3 | 14 |
| 2013/4/23 | 22:07:16 | 103.0 | 30.3 | 19 | 4.1 | 8 |
| 2013/5/1 | 2:14:15 | 102.9 | 30.2 | 14 | 4.3 | 17 |
| 2013/5/11 | 7:30:08 | 102.8 | 30.3 | 24 | 4.0 | 14 |
| 2013/5/11 | 8:50:39 | 103.0 | 30.3 | 19 | 4.3 | 11 |
| 2013/5/11 | 9:07:21 | 103.0 | 30.3 | 20 | 4.0 | 8 |
| 2013/5/15 | 20:29:11 | 105.2 | 32.5 | 10 | 4.4 | 4 |
| 2013/5/16 | 1:24:55 | 103.0 | 30.4 | 21 | 4.2 | 6 |
| 2013/5/26 | 21:01:59 | 103.0 | 30.3 | 17 | 4.1 | 17 |
| 2013/6/4 | 22:35:26 | 74.8 | 39.9 | 6 | 4.3 | 3 |
| 2013/7/6 | 5:09:54 | 102.9 | 30.3 | 20 | 4.0 | 6 |
| 2013/7/8 | 7:39:14 | 103.6 | 31.3 | 21 | 4.5 | 5 |
| 2013/7/21 | 16:35:17 | 100.8 | 22.9 | 12 | 4.1 | 3 |
| 2013/7/22 | 7:45:57 | 104.2 | 34.5 | 15 | 6.7 | 3 |
| 2013/7/27 | 0:25:05 | 104.9 | 33.0 | 10 | 4.5 | 4 |
| 2013/8/8 | 2:51:43 | 103.2 | 31.7 | 21 | 4.7 | 6 |
| 2013/8/28 | 20:57:41 | 85.8 | 42.1 | 26 | 4.2 | 5 |
| 2013/8/30 | 13:27:28 | 87.6 | 43.8 | 12 | 5.1 | 26 |
| 2013/9/20 | 5:37:01 | 101.5 | 37.7 | 15 | 5.3 | 3 |
| 2013/10/14 | 11:19:00 | 102.8 | 28.0 | 18 | 4.6 | 3 |
| 2013/10/15 | 17:22:27 | 101.4 | 23.1 | 14 | 4.3 | 3 |
| 2013/10/30 | 21:53:06 | 100.7 | 23.7 | 10 | 4.0 | 3 |
| 2013/11/4 | 16:04:37 | 75.5 | 39.8 | 20 | 4.3 | 5 |
| 2013/11/7 | 4:26:25 | 102.9 | 30.3 | 19 | 4.2 | 6 |
| 2013/11/16 | 23:36:41 | 103.0 | 26.4 | 10 | 4.6 | 6 |
| 2013/11/23 | 13:44:11 | 120.0 | 37.1 | 10 | 4.5 | 3 |
| 2013/11/28 | 16:23:54 | 100.6 | 25.4 | 10 | 4.7 | 7 |
| 2013/12/25 | 14:29:05 | 104.9 | 32.3 | 19 | 4.0 | 4 |
| 2013/12/29 | 8:29:11 | 102.0 | 29.9 | 16 | 4.0 | 3 |
| 2014/1/15 | 3:17:47 | 101.2 | 26.9 | 33 | 4.5 | 6 |
| 2014/1/28 | 20:01:59 | 101.2 | 22.5 | 7 | 4.7 | 7 |
| 2014/2/22 | 12:56:16 | 102.3 | 37.6 | 6 | 4.5 | 13 |
| 2014/4/2 | 14:04:43 | 103.6 | 31.2 | 15 | 4.2 | 6 |
| 2014/4/5 | 6:40:33 | 103.6 | 28.1 | 13 | 5.1 | 5 |
| 2014/4/11 | 10:30:58 | 103.2 | 31.7 | 22 | 4.9 | 7 |
| 2014/5/7 | 22:11:41 | 101.9 | 25.5 | 13 | 4.7 | 3 |
| 2014/6/10 | 7:54:16 | 105.2 | 32.6 | 15 | 4.8 | 6 |
| 2014/7/9 | 5:52:49 | 78.2 | 39.3 | 10 | 5.1 | 3 |
| 2014/8/3 | 16:30:12 | 103.3 | 27.1 | 10 | 6.6 | 7 |
| 2014/8/3 | 19:07:21 | 103.4 | 27.1 | 21 | 4.1 | 3 |
| 2014/8/3 | 21:47:10 | 103.4 | 27.1 | 10 | 4.1 | 3 |
| 2014/8/3 | 22:28:31 | 103.3 | 27.1 | 8 | 4.2 | 5 |
| 2014/8/4 | 3:30:32 | 103.4 | 27.1 | 5 | 4.4 | 5 |
| 2014/8/9 | 6:53:42 | 104.1 | 31.8 | 20 | 4.2 | 8 |
| 2014/8/10 | 12:39:12 | 103.4 | 27.0 | 10 | 4.0 | 4 |
| 2014/8/17 | 6:08:00 | 103.5 | 28.1 | 7 | 5.2 | 8 |
| 2014/8/17 | 17:11:47 | 103.6 | 28.1 | 13 | 4.2 | 3 |
| 2014/8/21 | 12:11:02 | 100.5 | 25.9 | 12 | 4.2 | 7 |
| 2014/8/27 | 18:45:13 | 101.0 | 23.0 | 10 | 4.0 | 6 |
| 2014/9/6 | 18:37:42 | 115.4 | 40.3 | 20 | 4.3 | 11 |
| 2014/9/10 | 16:59:30 | 103.4 | 27.1 | 17 | 4.3 | 5 |
| 2014/10/1 | 9:23:29 | 102.7 | 28.4 | 10 | 5.2 | 9 |
| 2014/10/7 | 21:49:39 | 100.6 | 23.4 | 10 | 6.9 | 5 |
| 2014/10/8 | 5:14:06 | 102.9 | 30.1 | 18 | 4.2 | 9 |
| 2014/10/25 | 10:26:02 | 102.4 | 34.3 | 15 | 4.7 | 3 |
| 2014/10/27 | 0:02:30 | 103.3 | 27.1 | 12 | 4.2 | 5 |
| 2014/11/22 | 16:55:28 | 101.7 | 30.3 | 20 | 6.4 | 4 |
| 2014/11/25 | 23:19:10 | 101.8 | 30.2 | 16 | 5.9 | 9 |
| 2014/12/6 | 2:43:45 | 100.5 | 23.3 | 10 | 5.9 | 4 |
| 2014/12/6 | 18:20:00 | 100.5 | 23.3 | 10 | 5.9 | 4 |
| 2014/12/21 | 13:09:37 | 101.5 | 27.9 | 11 | 4.1 | 5 |
| 2015/1/10 | 14:50:57 | 77.3 | 40.2 | 10 | 5.0 | 4 |
| 2015/1/14 | 13:21:00 | 103.2 | 29.3 | 14 | 5.0 | 6 |
| 2015/2/15 | 17:38:46 | 74.9 | 39.5 | 6 | 4.4 | 4 |
| 2015/2/22 | 14:42:56 | 85.7 | 44.1 | 4 | 5.1 | 6 |
| 2015/3/9 | 17:59:42 | 103.1 | 25.3 | 12 | 4.5 | 28 |
| 2015/6/5 | 18:30:46 | 102.8 | 30.3 | 18 | 4.0 | 9 |
| 2015/8/4 | 12:25:27 | 122.4 | 40.5 | 8 | 4.3 | 4 |
| 2015/8/20 | 6:04:36 | 84.4 | 42.0 | 7 | 4.2 | 5 |
| 2015/9/23 | 2:01:35 | 105.4 | 32.6 | 11 | 4.0 | 3 |
| 2015/10/24 | 19:29:40 | 102.1 | 29.1 | 16 | 4.0 | 6 |
| 2015/11/14 | 0:55:06 | 100.5 | 23.3 | 6 | 4.6 | 3 |
| 2015/11/23 | 5:02:42 | 100.4 | 38.0 | 10 | 5.2 | 4 |

**List of events from validating dataset in the period of 2016-2017**

| Event date | Time | Long. | Lat. | Depth(km) | Ms | Num. of records |
| --- | --- | --- | --- | --- | --- | --- |
| 2016/5/18 | 0:48:47 | 99.5 | 26.1 | 15 | 5.0 | 6 |
| 2017/3/12 | 20:21:00 | 103.4 | 27.1 | 10 | 4.5 | 5 |
| 2017/3/27 | 7:55:00 | 99.8 | 25.9 | 12 | 5.1 | 8 |
| 2017/8/8 | 21:19:46 | 103.8 | 33.2 | 20 | 7.0 | 3 |
| 2017/8/9 | 9:22:14 | 103.8 | 33.2 | 21 | 4.2 | 3 |
| 2017/8/9 | 9:32:48 | 103.7 | 33.3 | 16 | 4.3 | 3 |
| 2017/8/9 | 10:17:02 | 103.9 | 33.2 | 26 | 4.8 | 4 |
| 2017/8/10 | 3:02:12 | 103.8 | 33.3 | 22 | 4.1 | 4 |
| 2017/8/10 | 5:05:54 | 103.8 | 33.2 | 26 | 4.3 | 7 |
| 2017/8/10 | 17:48:34 | 103.8 | 33.2 | 26 | 4.1 | 6 |
| 2017/8/13 | 22:38:32 | 103.9 | 33.1 | 16 | 4.0 | 7 |
| 2017/9/30 | 14:14:00 | 105.0 | 32.3 | 13 | 5.4 | 9 |
| 2017/11/10 | 13:03:00 | 104.0 | 31.5 | 19 | 4.2 | 8 |

**Table S2. Coefficients for Pd^10km^ magnitude scaling relations**

| PTW | log_10_(Pd^10km^) =A+BM | | |  | M =A’+ B’log_10_(Pd^10km^) | | |
| --- | --- | --- | --- | --- | --- | --- | --- |
|  | A±ΔA | B±ΔB | Std. dev. |  | A’ | B’ | Std. dev. |
| 2s | -3.988±0.224 | 0.557±0.048 | 0.261 |  | 7.164 | 1.797 | 0.469 |
| 3s | -3.842±0.225 | 0.568±0.048 | 0.263 |  | 6.763 | 1.760 | 0.463 |
| 4s | -3.749±0.227 | 0.599±0.048 | 0.264 |  | 6.261 | 1.670 | 0.442 |
| 5s | -3.829±0.219 | 0.636±0.047 | 0.255 |  | 6.025 | 1.573 | 0.402 |
| 6s | -3.915±0.214 | 0.654±0.046 | 0.250 |  | 5.985 | 1.529 | 0.382 |
| 7s | -4.009±0.222 | 0.666±0.047 | 0.259 |  | 6.022 | 1.502 | 0.389 |
| 8s | -4.176±0.219 | 0.691±0.047 | 0.256 |  | 6.040 | 1.446 | 0.370 |
| 9s | -4.255±0.217 | 0.702±0.046 | 0.253 |  | 6.065 | 1.425 | 0.361 |
| 10s | -4.319±0.218 | 0.711±0.047 | 0.254 |  | 6.073 | 1.406 | 0.357 |

Table S3. Thresholds for M = 6.5

| PTW | 2s | 3s | | 4s | | 5s | |  | | 6s | | 7s | | 8s | | 9s | | 10s | |  |
| --- | --- | --- | --- | --- | --- | --- | --- | --- | --- | --- | --- | --- | --- | --- | --- | --- | --- | --- | --- | --- |
| Pd^10km^ | 0.234 | | 0.387 | | 0.756 | |  | | 1.114 | | 1.222 | | 1.146 | | 1.153 | | 1.127 | | 1.120 | |
| τ_c_ | 1.005 | | 1.018 | | - | |  | | - | | - | | - | | - | | - | | - | |

Table S4. Weights for magnitude estimation

|  | Predicted magnitude for M8 | | Underestimation error for M8 | | Weight | |
| --- | --- | --- | --- | --- | --- | --- |
| PTW_i_ | τ_c_ | P_d_^10km^ | τ_c_ | P_d_^10km^ | τ_c_ | P_d_^10km^ |
| 2 | 7.56 | 6.28 | -0.44 | -1.72 | 0.796 | 0.204 |
| 3 | 7.38 | 6.29 | -0.62 | -1.71 | 0.734 | 0.266 |
| 4 | — | 6.59 | — | -1.41 | 0.695 | 0.305 |
| 5 | — | 7.07 | — | -0.93 | 0.599 | 0.401 |
| 6 | — | 7.18 | — | -0.82 | 0.570 | 0.430 |
| 7 | — | 7.22 | — | -0.78 | 0.558 | 0.442 |
| 8 | — | 7.72 | — | -0.28 | 0.311 | 0.689 |
| 9 | — | 7.72 | — | -0.28 | 0.311 | 0.689 |
| 10 | — | 7.79 | — | -0.21 | 0.253 | 0.747 |
